# Comparison of postoperative complications in mediastinal lymph node dissection versus mediastinal lymph node sampling for early stage non-small cell lung cancer: Protocol for a systematic review and meta-analysis

**DOI:** 10.1371/journal.pone.0298368

**Published:** 2024-02-20

**Authors:** Qiao Chen, Weijuan Li, Ningning Cai, Weiwei Chen, Xiaojuan Zhao, Xiongfeng Huang

**Affiliations:** 1 Jiangxi University of Traditional Chinese Medicine, Nanchang, China; 2 Fuzhou Medical College of Nanchang University, Fuzhou, China; European Institute of Oncology: Istituto Europeo di Oncologia, ITALY

## Abstract

**Introduction:**

Lung cancer is the primary cause of cancer-related deaths worldwide, with high rates of morbidity and mortality. The most effective treatment for early stage (I-II) non-small cell lung cancer (NSCLC) is surgical resection. However, the extent of mediastinal lymph nodes removal required and the impact of their removal remains controversial. This systematic review and meta-analysis aimed to evaluate the postoperative complications in patients with stage I-II NSCLC who received mediastinal lymph node dissection (MLND) or mediastinal lymph node sampling (MLNS).

**Methods and analysis:**

According to the predefined inclusion criteria, we will conduct a comprehensive search for randomized controlled trials (RCTs) and observational studies examining the postoperative complications of MLND compared to MLNS in patients with stage I-II NSCLC. The search will be performed across multiple databases including PubMed, Embase, the Cochrane Library, CNKI, WanFang, Sinomed, VIP, Duxiu, and Web of Science from inception to February 2024. Additionally, relevant literature references will be retrieved and hand searching of pertinent journals will be conducted. Screening, data extraction, and quality assessment will be performed by two independent reviewers. Review Manager 5.4 will be applied in analyzing and synthesizing. The Grading of Recommendations Assessment, Development and Evaluation (GRADE) will be used to assess the quality of evidence for the whole RCTs and used Newcastle-Ottawa scale to assess the methodologic quality of observational studies.

**Ethics and dissemination:**

This study did not include personal information. Ethical approval was not required for this study. This study is based on a secondary analysis of the literature, so ethical review approval is not required. The final report will be published in a peer-reviewed journal.

**Conclusion:**

This systematic review will contribute to compare the safety and survival benefits of these two surgical techniques for the treatment of early stage NSCLC, to further guide the selection of surgical approaches.

**Trial registration:**

The protocol of the systematic review has been registered on Open Science Framework, with a registration number of DOI https://doi.org/10.17605/OSF.IO/N2Y5D.

## Introduction

Lung cancer is the primary cause of cancer-related deaths worldwide, with high rates of morbidity and mortality [1]. More than 1.2 million lung cancer patients die annually worldwide. In 2023, the American Cancer Society estimated that there will be about 238,340 new cases of lung cancer in the U.S. and about 127,070 people will die due to this disease [2]. The most common form of the disease is non–small cell lung cancer (NSCLC), which accounts for approximately 85% of lung cancers, and 5-year survival across all stages of the disease is approximately 14% [3].

The standard treatment for patients with early stage (I-II) NSCLC is surgery combined with neoadjuvant therapy; nonetheless, metastatic recurrence is still relatively common [4]. The primary metastatic route of lung cancer is through lymph node metastasis. Therefore, the typical surgical procedure for lung cancer is the removal of the primary tumor along with lymph node dissection, which also provides an accurate pathological staging of the cancer after surgery, forming the foundation for prognostication of the patient’s condition and determination of further treatment methods. Regional lymph node metastasis is a crucial factor impacting the early stage of NSCLC. However, the extent of mediastinal lymph nodes removal required and the impact of their removal remains controversial [5–8]. Some indications complete ipsilateral mediastinal lymph node dissection (MLND) is important for accurate staging and better locoregional control due to resection of otherwise undetected micrometastases [9–11]. Nevertheless, some arguments still exist against MLND including causing more complications, prolonging hospitalization and increasing mortality compared to mediastinal lymph node sampling (MLNS), one potential explanation may be that MLND requires a more extensive mediastinal dissection [12, 13]. However, limited data are available from studies to support it. Thus, it remains unclear whether or not MLND causes more postoperative complication rates when compared to MLNS.

For these reasons, we will carry out a systematic review and meta-analysis of existing randomized controlled trials (RCTs) and observational studies to evaluate the postoperative complications in patients with stage I-II NSCLC who received MLND or MLNS.

## Methods and analysis

This systematic review protocol was preregistered in the Open Science Framework Registries: https://doi.org/10.17605/OSF.IO/N2Y5D. This systematic review protocol follows the guidelines recommended in Preferred Reporting Items for Systematic Review and Meta-Analysis (PRISMA-P) [14]. The PRISMA-P checklist is attached as S1 Checklist.

### Inclusion and exclusion criteria

#### Include

1. **Population**. Stage I-II NSCLC.
2. **Exposures**. The control group employed MLNS, while the experimental group implemented MLND.
3. **Outcome**. The primary indicator was the incidence of postoperative complications including air leak, arrhythmia, chylothorax, pneumonia, recurrent laryngeal nerve lesions and the duration of postoperative chest drainage. The secondary indicators were intraoperative blood loss, and length of hospital stay.
4. **Study design**. RCTs or observational studies.

#### Exclusion

(1) Research experiments that are not rigorously designed; (2) Relevant and usable data cannot be obtained from the literature; (3) Trials in which interventions are interfered with by other treatments; (4)There are obvious errors in the data results.

### Database and retrieval strategy

Eligible studies will be identified by searching the relevant articles published in the following databases: PubMed, Embase, the Cochrane Library, CNKI, WanFang, Sinomed, VIP, Duxiu, and Web of Science from inception to February 2024. There will be no restriction on origin and languages. Search terms included: “mediastinal lymph node dissection”, “MLND”, “mediastinal lymph node sampling”, “MLNS”, “postoperative complications”, ‘‘non-small cell lung cancer” and ‘‘NSCLC”. In addition to our traditional database searches, we will also conduct a search for grey literature using Google Scholar. The reference list of all selected articles will be independently screened to identify additional studies left out in the initial search. Furthermore, our next step will be to review the reference lists of relevant articles to identify any grey literature that may have been missed in our initial search. To acquire comprehensive data for eligible studies, we will reach out to key authors and request any relevant information, including supplementary materials that may not have been fully disclosed or reported, as well as data from informal channels of relevant research.

### Study selection and data extraction

**Fig 1:**
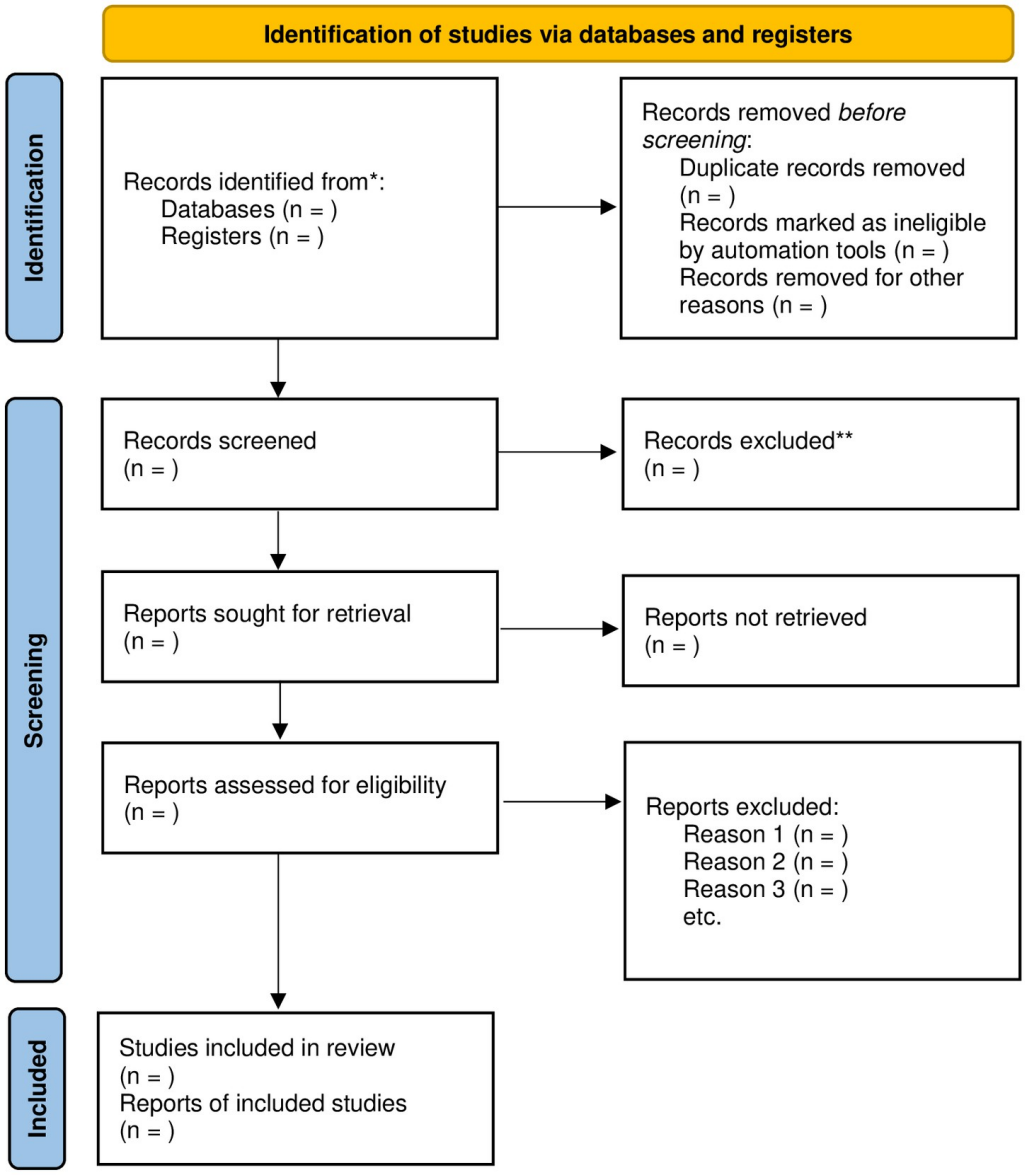
Flow diagram showing the selection process of articles. *Consider, if feasible to do so, reporting the number of records identified from each database or register searched (rather than the total number across all databases/registers). **If automation tools were used, indicate how many records were excluded by a human and how many were excluded by automation tools. *From*: Page MJ, McKenzie JE, Bossuyt PM, Boutron I, Hoffmann TC, Mulrow CD, et al. The PRISMA 2020 statement: an updated guideline for reporting systematic reviews. BMJ 2021;372:n71. doi: 10.1136/bmj.n71 For more information, visit: http://www.prisma-statement.org/.

Two reviewers will import the retrieved results of the searches into the Endnote X9 software and exclude duplicate studies. Then, they will screen the titles and abstracts to eliminate studies that do not meet the inclusion criteria. Finally, they will include the eligible studies after obtaining the full text of the remaining studies. If two reviewers have differing opinions, they will consult a third reviewer and make a final decision together. The process of study selection has been shown using a flow chart (Fig 1).

Finally, two authors will independently extract data using data extraction tables as recommended by the Cochrane Handbook for Systematic Reviews of Interventions. The following data will be extracted: study characteristics such as author, year of publication, country in which the study was conducted, study period, original inclusion criteria, and the total number of people included in the study; population characteristics such as mean age and time from diagnosis; intervention characteristics such as type, duration, frequency, etc. Any disagreements will be resolved by discussion until consensus is reached or by consulting a third researcher, the characteristics of the studies are attached as Table 1. We will also contact the original authors of papers via email or telephone if possible.

**Table 1:** General information of the included studies.

First author, year, country	Sample size		Participants	Intervention characteristics	Adjuvant treatment	Oncological results	Outcome assessment
	T(males/ females)	C(males/ females)					
							
							
							

### Assessment of study quality

Two reviewers will use the Cochrane Handbook for Systematic Reviews of Interventions to evaluate the risk of bias in the included studies. The evaluation criteria mainly encompassed the following seven areas: random sequence generation, allocation hiding, blinding of participants and personnel, blinding of outcome assessment, incomplete outcome data, selective outcome reporting, and other biases. The rating of the item’s quality will be based on three levels: high risk, unclear risk, or low risk [15]. The Grading of Recommendations Assessment, Development and Evaluation (GRADE) will be used to assess the quality of evidence for the whole RCTs [16]. We will use Newcastle-Ottawa scale to assess the methodologic quality of observational studies. The Newcastle-Ottawa Scale contains eight items that are categorized into three categories: selection (four items, one star each), comparability (one item, up to two stars), and exposure/outcome (three items, one star each) [17].

Two independent reviewers will assess the quality. Discussions and consensus with a senior investigator will resolve discrepancies between the two reviewers. The final results will be reviewed by two senior investigators.

### Data synthesis and analysis

#### Statistical analysis

Differences will be expressed as risk ratio (RR) with 95% confidence intervals (CI) for dichotomous outcomes. Heterogeneity across studies will be tested by using the *I*^2^ statistic, which is a quantitative measure of inconsistency across studies. Studies with an *I*^2^ statistic of 25% to 50% will be considered to have low heterogeneity, those with an *I*^2^ statistic of 50% to 75% will be considered to have moderate heterogeneity, and those with an *I*^2^ statistic of 75% to 100% will be considered to have high heterogeneity [18]. To further evaluate the extent of heterogeneity between publications, Cochran’s χ^2^ based Q statistic test will be employed. If the P value is greater than 0.10 of the Q-test, which indicates low heterogeneity or lack of heterogeneity among the included studies [19]. When heterogeneity is confirmed, the random-effect method will be used [20, 21]. Without statistically significant heterogeneity, the fixed-effect method will be used to combine the results [22, 23]. A two-sided P value of less than 0.05 was considered to indicate a statistically significant difference. All statistical analyses will be performed using Review Manager 5.4 provided by The Cochrane Collaboration.

#### Assessment of reporting biases

The funnel plot and Egger test will be used to assess reporting bias if there are 10 or more studies included in the meta-analysis. The trim and fill approach will be implemented to identify and address any asymmetries in the funnel resulting from publication bias, if appropriate.

#### Subgroup analysis

When heterogeneity is detected, subgroup analysis will be conducted to identify the source of heterogeneity.

#### Sensitivity analysis

For trials with sufficient data, sensitivity analyses will be conducted to assess the robustness and reliability of the findings. We will conduct sensitivity analysis based on heterogeneity, and sensitivity analysis may be conducted, and certain low-quality studies may be excluded when heterogeneity occurs.

## Discussion

Lung cancer is the primary cause of cancer-related deaths worldwide, with high rates of morbidity and mortality [1]. The overall survival rate for lung cancer patients is low, with only a 19.7% five-year survival rate [3]. Its pathological types include small cell lung cancer and non-small cell lung cancer, of which NSCLC accounts for about 80–85% [4]. With the aging of the population, the acceleration of urban industrialization, and the aggravation of environmental pollution, lung cancer has become a major threat to life and health, with incidence and mortality rates on the rise. Early detection of lung cancer, accurate staging, and appropriate treatment interventions are crucial to enhancing the survival prospects of patients.

At present, surgical resection of tumor and lymph node dissection is still one of the effective methods to treat lung cancer. Currently, the primary treatment protocol for NSCLC is the resection of the primary lesion along with complete lymph node dissection. However, complete lymph node dissection carries a wide surgical scope, high levels of trauma, and an augmented risk of postoperative complications and mortality, and it may result in a reduced quality of life. Therefore, there is still a lot of debate on whether a thorough lymph node dissection is necessary in clinical operation and practice for patients with early-stage NSCLC [24–28]. For these reasons, this study will perform a meta-analysis to assess the efficacy of MLND and MLNS in the treatment of early stage NSCLC patients. We will conduct a comprehensive analysis of the postoperative complications to compare the safety and survival benefits of these two surgical techniques for treating early stage NSCLC. However, this systematic evaluation still has some limitations, and we will try our best to rectify them. First, the current lack of a consistent standard for MLND and MLNS means that different surgical approaches may have potential impacts on patients’ postoperative conditions. To solve this problem, we will try our best to find consistent studies and address this limitation through subgroup analysis. Furthermore, we will include studies with various reconstruction techniques, which may introduce significant heterogeneity, and we will consider addressing this issue through randomization schemes and statistical analysis.

In conclusion, this study will use the existing literature research results to actively guide clinical practice, and promote the transformation of theoretical evidence into clinical practice. It is expected that the systematic review will contribute to compare the safety and survival benefits of these two surgical techniques for the treatment of early stage NSCLC, with the aim of further guiding the selection of surgical approaches.

## Supporting information

S1 Checklist PRISMA-P-checklist. (DOC)
